# Tumour necrosis factor gene polymorphism: a predictive factor for the development of post-transplant lymphoproliferative disease

**DOI:** 10.1038/sj.bjc.6605278

**Published:** 2009-09-08

**Authors:** K A McAulay, T Haque, D H Crawford

**Affiliations:** 1 Clinical and Basic Virology Laboratory, School of Biomedical Sciences, University of Edinburgh, Summerhall, Edinburgh EH9 1QH, UK; 2 Centre for Virology, UCL Medical School, Royal Free Campus, Rowland Hill Street, London NW3 2PF, UK

**Keywords:** PTLD, EBV, cytokine, polymorphism, TNF

## Abstract

**Background:**

Epstein–Barr virus-positive post-transplant lymphoproliferative disease (PTLD) is a potentially lethal complication of iatrogenic immunosupression after transplantation. Predicting the development of PTLD allowing early and effective intervention is therefore of importance. Polymorphisms within cytokine genes are implicated in susceptibility to, and progression of, disease however the published data are often conflicting. We undertook investigation of polymorphic alleles within cytokine genes in PTLD and non-PTLD transplant cohorts to determine risk factors for disease.

**Methods:**

SSP-PCR was used to analyse single nucleotide polymorphism within tumour necrosis factor (TNF)-*α*, interleukin- 1, -6, -10 and lymphotoxin-*α* genes. The TNF-*α* levels were measured by standard enzyme-linked immuno-absorbant assay.

**Results:**

We show an association between variant alleles within the TNF-*α* promoter (−1031C (*P*=0.005)); −863A (*P*=0.0001) and TNF receptor I promoter regions (−201T (*P*=0.02)); −1135C (*P*=0.03) with the development of PTLD. We also show an association with TNF-*α* promoter haplotypes with haplotype-3 significantly increased (*P*=0.0001) and haplotype-1 decreased (*P*=0.02) in PTLD patients compared to transplant controls. Furthermore, we show a significant increase (*P*=0.02) in the level of TNF-*α* in PTLD patient plasma (range 0–97.97 pg ml^−1^) compared to transplant controls (0–8.147 pg ml^−1^), with the highest levels found in individuals carrying the variant alleles.

**Conclusion:**

We suggest that genetic variation within TNF-*α* loci and the level of plasma cytokine could be used as a predictive risk factor for the development of PTLD.

Post-transplant lymphoproliferative disease (PTLD) is a potentially lethal complication of iatrogenic immunosupression after solid organ transplant (SOT) and bone marrow transplantation (Paya *et al*, 1999; Nalesnik, 2002). It occurs in up to 10% of all transplant recipients and in most cases is associated with Epstein–Barr virus (EBV) infection, a gammaherpes virus that infects around 90% of the adult population. On primary EBV infection the virus establishes a life-long asymptomatic infection in circulating B lymphocytes that are effectively controlled by EBV-specific cytotoxic T lymphocytes (CTLs). In transplant recipients, however, the immunosuppressive drug regimens used to control organ rejection, suppress the function of EBV-specific CTLs and can lead to the uncontrolled proliferation of EBV infected B lymphocytes and tumour formation. Reduction of immunosupression, radiotherapy, chemotherapy, surgery and monoclonal antibody-based regimens (Swinnen *et al*, 1995; Choquet *et al*, 2006) are routinely used to treat the disease on presentation of tumour, but even with such treatment options the mortality rate is approximately 50% (Opelz and Dohler, 2004; Choquet *et al*, 2007). More recently, infusions of *ex-vivo* expanded EBV-specific CTLs to selectively reconstitute EBV-specific immunity have proven useful in the treatment and prevention of EBV-positive PTLD (Savoldo *et al*, 2006; Haque *et al*, 2007). Strategies to predict the impending development of PTLD allowing early and effective intervention are therefore assuming increasing importance. A raised EBV viral load post transplant is one suggested marker of PTLD development (Riddler *et al*, 1994; Davis *et al*, 2004); however, the published data are contradictory with recent studies reporting that a high EBV viral load does not predict PTLD development post transplant (Benden *et al*, 2005; Sato *et al*, 2008; Wheless *et al*, 2008). More robust strategies are therefore required especially with the advent of promising new immunotherapies such as CTL infusion into the clinical setting.

Cytokine networks interact in a dynamic way to regulate the immune response, thus it is not surprising that variations in cytokine levels have been correlated with susceptibility to disease and disease progression (Brennan and McInnes, 2008; Feldmann, 2008). A fundamental issue of such studies is whether variation in the level of a secreted cytokine is the primary cause for the disease or a secondary downstream effect of the immune regulation process. Investigation of cytokine gene polymorphisms is one approach of unravelling this issue and may provide a genetic strategy for assigning a risk value to disease development.

Several studies have implicated polymorphisms within cytokine genes with the risk of symptomatic primary EBV infection (Hurme and Helminen, 1998; Helminen *et al*, 1999) and with the development of EBV-associated tumours. Interleukin (IL)-18 variants, for example, have been associated with more aggressive forms of nasopharyngeal carcinoma, whereas in contrast to a protective role in EBV-associated infectious mononucleosis (Helminen *et al*, 1999), the high producer IL-10 haplotype has been associated with EBV-positive gastric cancer (Wu *et al*, 2002; Pratesi *et al*, 2006). The low producer interferon gamma (IFN-*γ*) genotype has been implicated in EBV reactivation after stem cell transplantation and with the development of PTLD after renal and liver transplantation (VanBuskirk *et al*, 2001; Bogunia-Kubik *et al*, 2006; Lee *et al*, 2006). However, we recently investigated the low producer IFN-*γ* genotype in EBV-positive PTLD after various SOTs and found no association with the development of disease (Thomas *et al*, 2005). A more recent study investigating late-onset PTLD showed an association with tumour growth factor-*β*1 and IL-10 but not IFN-*γ* genotypes (Babel *et al*, 2007). The reasons for these observed differences are unknown but may, in part, be because of the difference in type of organ transplant, type of PTLD and/or small study cohorts.

Earlier studies have been criticised for use of small cohorts, unmatched controls and investigation of single polymorphic alleles (Ollier, 2004). Therefore, to resolve differences observed in the literature with regard to cytokine gene polymorphisms in EBV-positive PTLD, we analysed several cytokine gene and cytokine receptor gene polymorphisms (alleles, genotypes and haplotypes) in a large cohort of EBV-positive PTLD patients and appropriate controls to determine correlations, if any, with the development of disease.

## Materials and methods

### Study cohort

A cohort of EBV-associated PTLD patients was accrued as part of a phase II multi-centre clinical trial detailed in Haque *et al* (2007). Patients were recruited to the trial with informed written consent from patients or guardians. The study was approved by the Lothian Research Ethics Committee. Diagnosis of PTLD and its classification were determined by histological examination and EBV status determined by *in situ* hybridisation and immunohistocehemisrty methods (detailed in Haque *et al* (2007)). A blood sample was taken on diagnosis and plasma and peripheral blood mononuclear cells (PBMC) were collected.

An anonymised control cohort of EBV sero-positive heart transplant patients without any PTLD development was established from an earlier study investigating EBV infection in heart transplant patients (Hopwood *et al*, 2002). PBMC and plasma samples were prepared in a similar manner to PTLD patients.

Healthy EBV sero-positive individuals were recruited as part of an epidemiological study carried out at Edinburgh University. Details for enrolment and serotyping have been published elsewhere (Crawford *et al*, 2002, 2006). On recruitment, subjects provided a blood sample for EBV serology. PBMC and plasma samples were prepared as above.

### Cytokine polymorphism PCR

DNA was extracted using the Easy-DNA kit (Invitrogen, Paisley, UK) and amplified in 47 separate PCR reactions using primers amplifying polymorphic regions within tumour necrosis factor(TNF)-*α*, lymphotoxin (LT-*α*), IL-1-*α*, IL-6, IL-10, TNF receptor(R)I, TNFRII, IL-1R and IL-10R genes (Supplementary Table 1). Each primer mix also contained a set of control primers. Between 60 and 80 ng DNA were amplified in a reaction mix containing Bio*Taq* DNA polymerase and reagents (Bioline, London, UK): 1 × NH_4_ buffer; 1.5 mM MgCl_2_; 200 *μ*M dNTPs and 0.35 U Taq polymerase. Cycling parameters were staged as follows: 96°C for 1 min; 4 cycles of 96°C for 20 s, 75°C for 45 s, 72°C for 25 s; 20 cycles of 96°C for 25 s, 65°C for 50 s, 72°C for 30 s; 3 cycles of 96°C for 30 s, 55°C for 60 s, 72°C for 90 s; 5°C for 10 min. The resultant PCR product was visualised on a 2% agarose-ethidium bromide gel under UV illumination.

### Enzyme-linked immuno-absorbant assay (ELISA)

The level of human TNF-*α* was measured in plasma samples from PTLD patients, non-PTLD transplant control patients and healthy EBV sero-positive controls by quantitative ELISA (Mabtech, Nacka Strand, Sweden) following the manufacturer's instructions. Briefly, wells were coated overnight with the monoclonal antibody TNF-*α*-I, washed and blocked before the addition of quantified TNF standards and patient plasma (undiluted). Plasma was allowed to absorb for 2 h and was then washed. Bound TNF-*α* was detected using the biotinylated monoclonal antibody to TNF-*α*-II and streptavidin-alkaline phosphatase. The optical density at 405 nm was measured after 30 min incubation with the development substrate *p*-nitrophenyl-phosphate (Sigma, Gillingham, UK).

### Statistical methods

Each polymorphism was tested for Hardy–Weinberg disequilibrium by comparing the observed allele frequency with the expected frequency if equilibrium applied. Classical association analysis was conducted to compare allele frequencies between the groups of subjects (PTLD-positive and PTLD-negative post transplant cohorts; PTLD positive and healthy control cohorts) and tested using the Fisher's exact or *χ*^2^ test (this was not adjusted for multiple testing). The Mann–Whitney *U* test and the one-way ANOVA test were used to test for differences in the medians of quantitative variables. Tests were two-tailed and a *P*-value <0.05 was considered significant. All statistical analysis was performed using GraphPad PRISM software (GraphPad Software Inc., La Jolla, CA, USA).

## Results

Known polymorphic alleles/regions from the TNF-*α*, LT-*α*, IL-1-*α*, IL-6, IL-10, TNFRI, TNFRII, IL-1R and IL-10R (detailed in Supplementary Table 1) were analysed in transplant patients with (*n*=45) or without (*n*=65) EBV-positive PTLD (designated PTLD and transplant control subject groups, respectively). Similar analyses were also performed on a healthy adult EBV sero-positive control cohort (*n*=183). Both the transplant and healthy control cohorts were compared with published frequencies (HapMap Caucasian European (CEU) and CEU Geno frequencies; Pubmed SNP database www.Ncbi.nlm.nih.gov) to assess any variation from the normal expected frequencies: no major variation from expected frequencies was noted. All polymorphic alleles were found to be in Hardy–Weinberg equilibrium except for the single nucleotide polymorphism at position 230 (A/G base change) in the TNFRI promoter region within the transplant control group and therefore individual analysis of this allele was not performed.

### Increased frequency of the TNF promoter −1031C and −863A variant alleles in PTLD subjects

**Figure 1:**
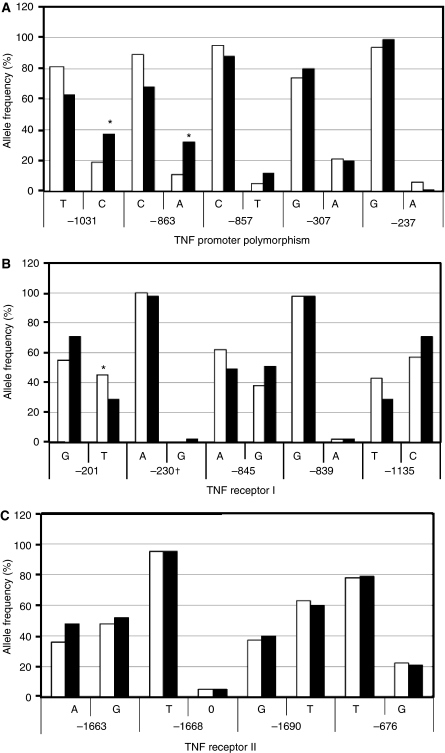
Allele frequencies of TNF promoter polymorphisms in transplant patients with and without PTLD. Allele frequencies (%) were calculated for each polymorphic allele and statistical analysis performed using Fisher's exact two-sided tests (significant *P*-value, *P*<0.05). Black bars (▪) represent PTLD transplant patients and white bars (□) transplant control patients. Significant *P*-value highlighted with asterisk (^*^). (**A**) Polymorphic alleles from the TNF-*α* promoter; (**B**) polymorphic alleles from TNF receptor I; (**C**) polymorphic alleles from TNF receptor II.

A total of five polymorphic alleles within the TNF promoter region (nucleotide positions relative to transcription start site −1031(T/C), −863(C/A), −857(C/T), −307(G/A) and −237(G/A)) were investigated. For the TNF promoter polymorphism at position −1031(T/C), we observed a significant increase in the frequency of the rarer TNF −1031C allele in the PTLD subject group compared with the non-PTLD transplant control group (37% *vs* 19%; *P*=0.005; Figure 1A; Table 1) and when compared with the healthy control group (37% *vs* 23%; *P*=0.01; Table 1). A significant difference was also found in the genotype frequency of this polymorphism with homozygous CC and heterozygous TC frequencies increased whereas homozygous TT frequencies were decreased (CC: 9% PTLD subjects *vs* 3% transplant control subjects; TC: 38% *vs* 32%; TT: 44% *vs* 65%, *P*=0.01; CC: 3%, TC: 40%, TT: 57%; *P*=0.001 for healthy control subjects, Table 2).

Likewise for position TNF-863(C/A) we observed a significant increase in the TNF-863A allele frequency in the PTLD transplant group compared with the transplant control group (32% *vs* 11%; *P*=0.0001; Figure 1A; Table 1) and when compared with the healthy control group (32% *vs* 15%; *P*=0.0004; Table 1). Comparison of the genotype frequencies between each group also showed a significant difference for this polymorphism (CC: 44% PTLD subjects *vs* 82% transplant control subjects; CA: 47% *vs* 15%; AA: 9% *vs* 3%; *P*=0.0003. CC: 73%, CA: 25%, AA: 2%, *P*=0.0007; Table 2). The remaining investigated TNF promoter polymorphisms, at nucleotide positions −857(C/T), −307(G/A) and −237(G/A), showed no differences in allele or genotype frequency between transplant patients with or without PTLD or healthy controls (Table 2).

From the investigated TNF promoter polymorphisms it is possible to assign one out of six TNF promoter haplotypes, detailed in Table 3 (Grutters *et al*, 2002). Haplotype-1 (TCCGG) was under represented within the PTLD transplant group compared with the transplant control group (62% *vs* 83%, *P*=0.02; OR=2.6 (95% CI: 1.2–7.29)) whereas haplotype-3 (CACGG) was over represented (50% *vs* 14%, *P*=0.0001; OR=0.16 (95% CI: 0.06–0.4; Table 3). Haplotype-3 remained significantly over represented when compared with healthy controls (50% *vs* 26%, *P*=0.003; Table 3). Haplotypes-2, -4, -5 and -6 were comparable between all groups (Table 3).

### Decreased frequency of the TNFRI promoter −201T and increased frequency of −1135C alleles in PTLD subjects

Several polymorphisms within the TNFRII locus (exon-10 nucleotide position +1663(A/G), +1668(T/G), +1690(C/T) and exon-6, position +676(T/G)) and TNFRI promoter regions (nucleotide positions −201(G/T), −230(A/G), −845(A/G), −839(G/A), −1135(T/C)) were investigated. Analysis of the TNFRI promoter at position −201 showed a significant decrease in the frequency of the −201T allele within the PTLD group compared with the transplant control group (45% *vs* 29%, *P*=0.02; Figure 1B; Table 1). This was also true when the PTLD cohort was compared with the healthy control group (47% *vs* 29%, *P*=0.003; Table 1). The TNFRI promoter −1135C allele was significantly increased in the PTLD group compared with the transplant control group (71% *vs* 57%, *P*=0.03; Figure 1B; Table 1) and also when compared with the healthy control group (71% *vs* 53%, *P*=0.002; Table 1). The TNFR1 promoter −845G allele was significantly increased in the PTLD transplant group when compared with the healthy control group but not when compared with the transplant control group (51% *vs* 36%, *P*=0.01; Table 1). No differences were observed for TNFRII positions −1663, −1668, −1690, −676 or TNFRI promoter positions −230, −845 and −839 (Figure 1B and C; Table 1).

Genotype frequencies of TNF receptors I and II polymorphisms were also compared between the PTLD and transplant control groups. Analysis of the TNFRI promoter at position −201 showed a significant increase in the frequency of the −201GG genotype and a decrease of the −201TT genotype in the PTLD group compared with the transplant and healthy control groups (GG: 47% *vs* 31%, TT: 4% *vs* 20%, *P*=0.03 for PTLD *vs* transplant controls; GG: 47% *vs* 26%, TT: 4% *vs* 20%, *P*=0.006 for PTLD *vs* healthy controls; Table 2). Similar analysis of the TNFRI promoter position −1135 resulted in an observed decrease in the frequency of the genotype −1135TT (4% *vs* 17%) and an increase in the frequency of genotype −1135CC (47% *vs* 31%) within the PTLD group compared with the transplant control group, however, this did not reach significance (*P*=0.06; Table 2). Significance was reached when the PTLD transplant group was compared with healthy controls (TT: 4% *vs* 20%, CC: 47% *vs* 25%, *P*=0.005; Table 2). A significant decrease in the GG genotype of −845 was also noted between the PTLD transplant group and healthy controls (22% *vs* 9%, *P*=0.02; Table 2). No genotypic differences were observed for TNFRII positions −1663, −1668, −1690, −676 or for TNFRI promoter positions −230, −845 and −839 (Table 2).

Determination of the TNFRI promoter haplotypes from nucleotide positions −201, −230 and −845 results in five possible haplotypes, detailed in Table 4. An increase in the frequency of haplotype-1 (GAG) was observed in the PTLD group compared with the transplant control group (80% *vs* 63%) and a decrease in the frequency of haplotype-3 (TAA: 53% *vs* 69%, Table 4). However, these differences did not reach statistical significance (*P*=0.05 and 0.11, respectively). However, both haplotypes-1 and -3 were significantly different when the PTLD cohort was compared with the healthy control cohort (GAG: 80% *vs* 63%, *P*=0.03; TAA: 53% *vs* 74%, *P*=0.01; Table 4).

No difference in frequency was observed for any of the polymorphic alleles, genotypes or haplotypes investigated from the LT-*α*, IL-1, IL-6 and IL-10 loci and for those alleles from the IL-1R1 and IL-10RI loci.

### Increased plasma levels of TNF-*α* in patients with EBV-positive PTLD

**Figure 2:**
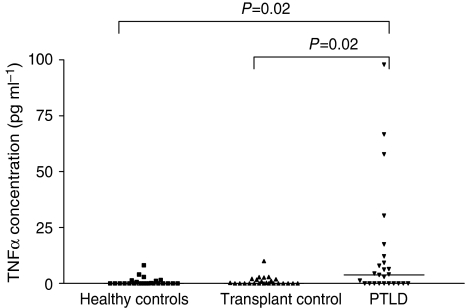
TNF-*α* levels in plasma from healthy controls and transplant patients with and without PTLD. The TNF-*α* level in plasma was measured by ELISA and the concentration estimated in relation to a set of known standards. Median levels are highlighted by the black bars and statistical analysis performed using the Mann–Whitney *U* test (significant *P*-value, *P*<0.05).

**Figure 3:**
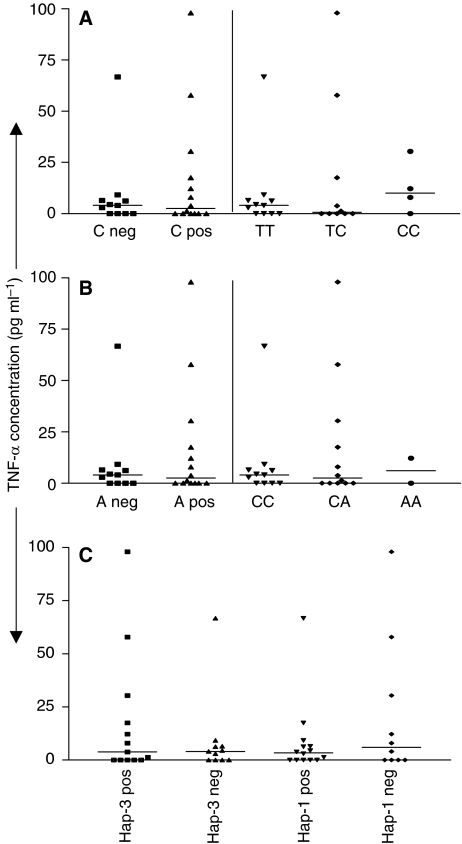
TNF-*α* levels in plasma from PTLD patients in relation to genetic variance. The level of TNF-*α* was estimated and assessed in relation to variant allele status (negative or positive), genotype or haplotype for position –1031(T/C) (**A**), −863(C/A) (**B**) and for the TNF-*α* promoter haplotype (**C**). Median levels are highlighted by the black bars and statistical analysis performed using the Mann–Whitney *U* test or one-way ANOVA test (significant *P*-value, *P*<0.05).

Polymorphisms within the TNF-*α* promoter regions have been associated with variation in the level of cytokine produced by cells (Koss *et al*, 2000). We therefore measured the levels of TNF-*α* in the plasma of our patient groups EBV-positive PTLD (*n*=25) and non-PTLD transplant controls (*n*=25) as well as in a cohort of healthy EBV sero-positve controls (*n*=25). Similar plasma TNF-*α* levels were observed for the healthy EBV sero-positve and non-PTLD transplant control groups (range 0–8.147 pg ml^−1^, median 0; and 0–9.981 pg ml^−1^, median 0, respectively). In contrast, there was a significant increase in the level of TNF-*α* in plasma obtained from PTLD patients (range 0–97.97 pg ml^−1^, median 3.801; *P*=0.02; Figure 2). We next assessed the TNF-*α* levels in relation to the polymorphic alleles and genotype of the PTLD patient group. For the TNF promoter polymorphism at position −1031(T/C), we assessed those with (C positive) and without (C negative) the risk allele. A median value of 4.053pg ml^−1^ (range 0–66.69) was observed for C-negative individuals compared with a median of 2.547 pg ml^−1^ (range 0–97.97) for C-positive individuals, however this was not a significant difference (Figure 3A). Similarly, we compared levels between the three genotypes and found no significant differences in TNF-*α* levels (Figure 3A). No significant difference in TNF-*α* levels for alleles and genotypes was observed for TNF promoter polymorphisms at position −863(C/A), TNF promoter haploytpes -1 and -3, or TNF receptor loci −201(G/T) and −1135(T/C) (Figures 3B, 3C; Table 5).

## Discussion

In this study, we have assessed polymorphisms within the TNF, LT-*α*, IL-1-*α*, IL-6 and IL-10 loci and their corresponding receptor loci for evidence of an association with the development of EBV-associated PTLD. The rarer alleles of the TNF promoter polymorphisms at nucleotide positions −1031(C allele) and −863(A allele) were found to be significantly increased in EBV-positive PTLD cases compared with non-PTLD transplant and healthy controls. Likewise, genotypes containing the non-ancestral allele were also significantly increased in the EBV-positive PTLD cases compared with transplant and healthy control groups. Furthermore, these polymorphic differences within the TNF promoter region resulted in a significant increase of TNF haplotype-3 (CACGG) and a decrease in TNF haplotype-1 (TCCGG) in EBV-positive PTLD subjects compared with transplant and healthy controls suggesting an important role for these haplotypes in determining susceptibility to EBV-positive lymphoma after transplantation. Interestingly, the frequency of the TNFRI promoter −1135C allele was also significantly increased and the −201T allele decreased in EBV-positive PTLD compared with transplant and healthy controls. A corresponding increase in the frequency of the homozygous −1135CC and a decrease in the −201TT genotypes were also observed for the EBV-positive PTLD group confirming the importance of the TNF family of cytokines and their receptors in the development of EBV-positive PTLD.

TNF is a member of the TNF family whose members function as potent mediators of immune regulation and inflammation. The TNF genes are located in the HLA class III region on chromosome 6p21.3 and are closely linked to the polymorphic HLA-B and -DR regions (Nedwin *et al*, 1985). TNF cytokines bind to two cellular receptors; TNFRI, which is widespread in many cells types and activated by soluble ligand, and the TNFRII that is primarily expressed on haemopoietic cells (Chan *et al*, 2000; Locksley *et al*, 2001). Both receptors are also shed and act as competitive soluble TNF-binding proteins consequently affecting the levels of TNF. In the case of TNF, ligand-receptor binding leads to the recruitment of intracellular adaptor proteins that activate several signal transduction pathways including the transcription factor NF-*κ*B and the apoptotic pathway through capsase 8 (Balkwill, 2006). Many pathological situations are determined by the balance between such survival and apoptotic signals. Therefore, gene polymorphisms that alter this signalling process either through the ligand or through the receptor are important.

The function of the TNF-863A variant highlighted in our PTLD cohort has been widely investigated. The nucleotide change from C to A has been shown to have a clear effect on the binding of the NF-*κ*B transcription complex to its DNA-binding domain. In particular, the affinity of the NF*κ*B p50–p50 heterodimer, which acts as a transcriptional repressor when bound to the TNF promoter, is significantly decreased for the −863A variants (Udalova *et al*, 2000). Decreased binding is thought to result in inadequate downregulation of TNF gene expression and therefore increased TNF production. As yet, there is no comparable molecular data for the TNF-1031C allele (however there is some degree of linkage between the −1031 and −863 alleles) or for the TNFRI promoter −201T and −1135C alleles.

Analysis of TNF-*α* levels in plasma from our cohorts showed a significant difference in levels between transplant controls and EBV-positive PTLD patients in general with higher TNF-*α* levels detected in the EBV-positive PTLD group. We did not detect a significant difference in TNF-*α* levels when we assessed the effect of the genetic variants within the PTLD cohort however there was a slight trend for higher TNF-*α* levels in the variant groups (Figure 3). This trend may become stronger with a larger cohort of PTLD individuals carrying the variant allele. Determining TNF-*α* levels in variant positive and negative individuals after transplant and before development of disease would provide further information on the predictive value of TNF-*α* levels.

Interestingly, the presence of the TNF-863A allele has also been positively associated with susceptibility to B-cell malignancies (non-Hodgkin's lymphomas) in the general population (Spink *et al*, 2006). Possession of specific alleles that act to increase TNF expression may therefore be central to the pathogenesis and susceptibility to lymphoid disease. Indeed, TNF-based mechanisms, such as direct DNA damage, anti-apoptotic activity and induction of cytokines, have been implicated in several cancers (Balkwill, 2006).

In summary, we have shown an association between variant alleles of the TNF promoter and the subsequent TNF promoter haplotype with the development of EBV-positive PTLD (although it has to be noted that this data set was not adjusted for multiple testing and so type 1 error cannot be completely ruled out). We have also shown that TNF-*α* levels are significantly higher in EBV-positive PTLD patients. However, there remains a group of EBV-positive PTLD patients who do not carry these alleles, genotypes or haplotypes, perhaps indicating that these polymorphisms are not independently functional and that other, as yet, unidentified variants are in linkage disequlibrium with these loci. These data require confirmation in a second, larger sized cohort to be certain of an association (power calculations based on this pilot study indicate a PTLD and non-PTLD transplant group size of 105 subjects would provide 80% power, 95% CI, OR=2.5) and further analysis of soluble TNF levels may offer some information on the functional activity of these polymorphic alleles. Nevertheless, the genotypic evidence for the involvement of TNF in EBV-positive PTLD presented here provides further information for identifying those most at risk.

## Figures and Tables

**Table 1:** Allele frequencies of the TNF-*á* promoter and TNF receptor polymorphisms in transplant patients with and without PTLD

**Polymorphism**	**Allele**	**Transplant subjects without PTLD (*n*=65) freq (%)**	**Transplant subjects with PTLD (*n*=45) freq (%)**	***P*-value^a^**	**Healthy control subjects (*n*=183) freq (%)**	***P*-valuea,^b^**
*TNF-α promoter*						
−1031	T	81	63	0.005^*^	74	0.02^*^
	C	19	37		23	
−863	C	89	68	0.0001^*^	85	0.004^*^
	A	11	32		15	
−857	C	95	88	0.08	91	0.43
	T	5	12		9	
−307	G	74	80	1	80	1
	A	21	20		20	
−237	G	94	99	0.08	95	0.14
	A	6	1		5	
						
*TNF receptor II*						
−1663	A	36	48	0.56	49	0.91
	G	48	52		51	
−1668	T	95	95	1	90	0.20
	G	5	5		10	
−1690	C	37	40	0.67	38	0.81
	T	63	60		62	
−676	T	78	79	1	71	0.18
	G	22	21		29	
						
*TNF receptor I promoter*						
−201	G	55	71	0.02^*^	53	0.003^*^
	T	45	29		47	
−230^b^	A	100	98	0.16	99	0.26
	G	0	2		1	
−845	A	62	49	0.07	64	0.01^*^
	G	38	51		36	
−839	G	98	98	1	98	0.66
	A	2	2		2	
−1135	T	43	29	0.03^*^	47	0.002^*^
	C	57	71		53	

Abbreviations: freq=frequency; TNF=tumour necrosis factor.

a Fisher's exact two-sided *P*-value (not adjusted for multiple testing).

b Comparison between post-transplant lymphoproliferative disease (PTLD) and healthy control groups; ^*^significant *P*-value, *P*<0.05.

**Table 2:** Genotype frequencies of the TNF promoter, TNF receptor II and TNF receptor I promoter polymorphisms in transplant patients with and without PTLD

**Polymorphism**	**Genotype**	**Transplant subjects without PTLD (*n*=65) freq (%)**	**Transplant subjects with PTLD (*n*=45) freq (%)**	***P*-value^a^**	**Healthy control subjects (*n*=183) freq (%)**	***P*-valuea,^b^**
*TNF-α promoter*						
−1031	TT	65	44	0.01^*^	57	0.001^*^
	TC	32	38		40	
	CC	3	9		3	
−863	CC	82	44	0.0003^*^	73	0.0007^*^
	CA	15	47		25	
	AA	3	9		2	
−857	CC	89	78	0.18	84	0.63
	CT	11	20		12	
	TT	0	20		2	
−307	GG	63	66	0.78	64	0.71
	GA	32	27		32	
	AA	5	7		4	
−237	GG	88	98	0.08	40	0.10
	GA	12	2		10	
	AA	0	0		0	
						
*TNF receptor II*						
−1663	AA	18	20	0.39	26	0.53
	AG	35	55		45	
	GG	31	25		29	
−1668	TT	91	90	0.89	80	0.14
	TG	9	10		20	
	GG	0	0		0	
−1690	CC	11	16	0.25	18	0.75
	CT	52	48		41	
	TT	37	36		41	
−676	TT	65	64	0.97	52	0.32
	TG	28	29		38	
	GG	8	7		10	
						
*TNF receptor I promoter*						
−201	GG	31	47	0.03^*^	26	0.006^*^
	GT	48	49		54	
	TT	20	4		20	
−230	AA	100	96	0.08	98	0.25
	AG	0	4		2	
	GG	0	0		0	
−845	AA	38	20	0.12	37	0.02^*^
	AG	48	58		53	
	GG	14	22		9	
−839	GG	95	96	0.96	97	0.73
	GA	5	4		3	
	AA	0	0		0	
−1135	TT	17	4	0.06	20	0.005^*^
	TC	52	49		54	
	CC	31	47		25	

Abbreviations: freq=frequency; TNF=tumour necrosis factor.

a Chi-square 3 × 2 contingency table (not adjusted for multiple testing).

b Comparison between post-transplant lymphoproliferative disease (PTLD) and healthy control groups; ^*^significant *P*-value, *P*<0.05.

**Table 3:** TNF promoter haplotypes in PTLD and control subjects

	**TNF promoter polymorphism**					**Transplant subjects without PTLD (*n*=64)**	**Transplant subjects with PTLD (*n*=42)**	**Odds ratio**		**Healthy control subjects (*n*=176)**	**Odds ratio**	
**Haplotype**	**−1031**	**−863**	**−857**	**−307**	**−237**	**freq %**	**freq %**	**(95% CI)**	***P*-value^a^**	**freq %**	**(95% CI)**	***P*-valuea,^b^**
1	T	C	C	G	G	83	62	2.96 (1.2–7.29)	0.02^*^	76	0.52 (0.26–1.07)	0.08
2	T	C	C	A	G	36	31	1.25 (0.54–2.87)	0.67	37	0.76 (0.37–1.56)	0.59
3	C	A	C	G	G	14	50	0.16 (0.06–0.4)	0.0001^*^	26	2.91 (1.46–5.8)	0.003^*^
4	T	C	T	G	G	9	19	0.43 (0.14–1.37)	0.23	15	1.38 (0.57–3.26)	0.48
5	C	C	C	G	A	14	2	6.70 (0.81–55.10)	0.08	9	0.24 (0.03–1.90)	0.2
6	C	C	C	G	G	3	7	0.41 (0.06–2.62)	0.38	8	0.89 (0.24–3.25)	1

Abbreviations: CI=confidence interval; freq=frequency; TNF=tumour necrosis factor.

Data are given as frequency with absolute numbers in parentheses.

a Fisher's exact two-sided *P*-value (not adjusted for multiple testing).

b Comparison between post-transplant lymphoproliferative disease (PTLD) and healthy control groups; ^*^significant *P*-value, *P*<0.05.

**Table 4:** TNF receptor I promoter haplotypes in PTLD

	**TNF receptor I promoter polymorphism**			**Transplant subjects without PTLD**	**Transplant subjects with PTLD**		**Healthy control subjects**	
**Haplotype**	**−201**	**−230**	**−845**	**freq (%)**	**freq (%)**	***P*-value^a^**	**freq (%)**	***P*-value^a,b^**
1	G	A	G	63	80	0.05	63	0.03^*^
2	G	A	A	31	33	0.83	29	0.59
3	T	A	A	69	53	0.11	74	0.01^*^
4	G	G	A	0	4	0.16	1	0.17
5	G	G	G	0	0	—	1	1

Abbreviations: freq=frequency; TNF=tumour necrosis factor. ^a^Fisher's exact two-sided *P*-value (not adjusted for multiple testing).

a Fisher's exact two-sided *P*-value (not adjusted for multiple testing).

b Comparison between post-transplant lymphoproliferative disease (PTLD) and healthy control groups;

^*^significant *P*-value, *P*<0.05.

**Table 5:** TNF-*α* levels in plasma of PTLD patients; genetic subgroup analysis

**Levels of TNF-*α* in Plasma**			
**Polymorphic loci**	**Number**	**Median (range, pg ml^−1^)**	***P*-value^a^**
*TNF 1031*			
C allele negative	11	4.053 (0–66.69)	0.85
C allele positive	14	2.547 (0–97.97)	
TT	11	4.053 (0–66.69)	0.56
TC	10	0.645 (0–97.97)	
CC	4	10.06 (0–30.34)	
			
*TNF 865*			
A allele negative	11	4.053 (0–66.69)	0.85
A allele positive	14	2.547 (0–97.97)	
CC	11	4.053 (0–66.69)	0.96
CA	12	2.547 (0–97.97)	
AA	2	6.088 (0–12.18	
			
*TNF haplotype*			
Haplotype-3 positive	13	3.801 (0–97.97)	0.68
Haplotype-3 negative	11	4.053 (0–66.69)	
Haplotype-1 positive	14	3.378 (0–66.69)	0.53
Haplotype-1 negative	10	5.997 (0–97.97)	
			
*TNFR 201*			
T allele negative	13	4.427 (0–97.97)	0.31
T allele positive	12	1.477 (0–30.34)	
GG	13	4.427 (0–97.97)	0.27
GT	10	0 (0–17.52)	
TT	2	16.64 (0–13.69)	
			
*TNFR 1135*			
C allele negative	2	16.64 (0–30.34)	—
C allele positive	23	3.801 (0–97.97)	
TT	2	16.64 (0–30.34)	0.27
TC	10	0 (0–17.52)	
CC	13	4.427 (0–97.97)	

a Mann–Whitney *U* Test or one-way ANOVA

(Significant *P*-value, *P*<0.05).
