# Supplementary material for: Tumour necrosis factor gene polymorphism: a predictive factor for the development of post-transplant lymphoproliferative disease
Source: Br J Cancer. 2009 Sep 8;101(6):1019–27. doi: 10.1038/sj.bjc.6605278 (PMC2743368; doi:10.1038/sj.bjc.6605278)
Supplement: Supplementary Table 1 [file 6605278x1.doc]

Supplementary Table 1: Single Nucleotide polymorphism location, position, base

change and population frequency

| **SNP** | **Cytokine location** | **Position** | **Base change** | **Population frequency^a^**  **(%)** |
| --- | --- | --- | --- | --- |
| rs1799964 | TNF promoter | -1031 | T/C | 79/21 |
| rs1800630 | TNF promoter | -863 | C/A | 84/16 |
| rs1799724 | TNF promoter | -857 | C/T | 93/7 |
| rs1800629 | TNF promoter | -308 | G/A | 78/22 |
| rs361525 | TNF promoter | -238 | G/A | 93/7 |
| rs4149570 | TNF receptor I promoter | -201 | G/T | 69/31 |
| rs4149621 | TNF receptor I promoter | -230 | A/G | 100/0 |
| rs767455 | TNF receptor I promoter | -845 | A/G | 49/51 |
| rs4149584 | TNF receptor I promoter | -839 | G/A | 98/2 |
| rs1800692 | TNF receptor I promoter | -1135 | T/C | 41/59 |
| rs1061624 | TNF receptor II exon 10 | -1663 | A/G | 51/48 |
| rs5030792 | TNF receptor II exon 10 | -1668 | T/G | nd |
| rs3397 | TNF receptor II exon 10 | -1690 | C/T | 47/53 |
| rs1061622 | TNF receptor II exon 6 | +676 | T/G | 75/25 |
| rs1041981 | LTα exon 3 | +720 | C/A | 64/36 |
| rs746868 | LTα intron 1 | +365 | C/G | 58/42 |
| rs909253 | LTα intron 1 | +249 | A/G | 64/36 |
| rs1800587 | IL-1α | -889 | C/T | 69/31 |
| rs2234650 | IL-1R1 | -1339 | C/T | nd |
| rs1800795 | IL-6 promoter | -174 | C/G | 53/47 |
| rs2069845 | IL-6 intron 4 | 3331 | A/G | 46/54 |
| rs1800896 | IL-10 promoter | -1082 | A/G | 47/53 |
| rs1800871 | IL-10 promoter | -819 | C/T | 83/17 |
| rs1800872 | IL-10 promoter | -592 | C/A | 80/20 |
| rs9610 | IL-10RA | -241 | G/A | 60/40 |

^a^ HapMap Caucasian European (CEU) population frequency or CEU Geno panel frequency (Pubmed

SNP database [www.ncbi.nlm.nih.gov](http://www.ncbi.nlm.nih.gov/));

nd: no data
